# Effectiveness of hyperbaric oxygen therapy in bone marrow edemas of the knee: A retrospective study

**DOI:** 10.1097/MD.0000000000033498

**Published:** 2022-04-07

**Authors:** Burak Ozturan, Muhlik Akyuerek

**Affiliations:** a Acibadem Kozyatagi Hospital, Istanbul, Turkey; b Maria-Josef-Hospital, Greven, Germany.

**Keywords:** bone marrow edema, hyperbaric oxygen, knee, MRI, treatment

## Abstract

Bone marrow edema (BME) is a self-limiting syndrome that can be caused by many pathological conditions. The most frequently seen symptom of BME is pain. Hyperbaric oxygen therapy (HBOT) is an available treatment. This study aims to present the clinical results of quantitatively evaluating the use of HBOT. We evaluated all BME patients 18 to 65 years old without osteoarthritis, inflammatory rheumatological disease, or malignancy diagnosed through magnetic resonance imaging. All were treated with acetylsalicylic acid (100 mg daily) and bisphosphonates (70 mg alendronate once a week) and were instructed to avoid weight-bearing activities. Some of the patients also received HBOT. We divided the patients into 2 groups: 1 group took HBOT; the other did not. We used the Wilcoxon test to compare groups. HBOT is an effective treatment option for BME. We quantitatively measured faster healing when HBOT was used for BME of the knee. There were no significant side effects.

## 1. Introduction

Bone marrow edema (BME) is a self-limiting syndrome that can be caused by many pathological conditions. Trauma, acute trauma, and recurrent chronic microtrauma are the primary causes of BME in the knee.^[1]^ Magnetic resonance imaging (MRI) provides the best viewing option for diagnosis: a low signal intensity in a T1-weighted MRI or a high signal intensity in a T2-weighted MRI.^[2,3]^

The most commonly seen symptom of BME is pain, which increases during weight-bearing activity and at night.^[4]^ It is caused by an increase in intraosseous pressure due to excess fluid in the bone.^[5,6]^ Many different diseases can underlie BME. Hofmann et al classify these diseases into 3 groups: ischemic BME, which includes osteonecrosis, osteochondritis diseases, and complex regional pain syndrome; mechanical BME, which includes bone contusions, microfractures, and stress fractures; and reactive BME, which includes gonarthrosis, osteoarthritis, and postoperative and tumor-related conditions.^[4]^

Conservative treatment is generally the first choice for care. Pain relief and avoidance of weight-bearing activities are essential.^[2]^ Other options include bisphosphonate, iloprost, vitamin D, extracorporeal shock waves, and pulsed electromagnetic fields. Surgical treatment, such as core decompression or subchondroplasty, maybe the next choice when conservative treatment is insufficient.^[2]^ Treatment with hyperbaric oxygen therapy (HBOT) is an alternative to conservative treatment. This study aims to present the clinical results of quantitatively evaluating the use of HBOT.

## 2. Method

The Istanbul Medeniyet University Ethical Committee approved this study on March 16, 2022.

Patients admitted to the hospital between January 1, 2018, and January 1, 2021, were evaluated retrospectively. Those who had osteoarthritis, inflammatory rheumatological disease, and malignancies were excluded. We evaluated all patients aged between 18 and 65 years old who had been diagnosed by MRI and treated for BME. From this group, we selected all patients suffering from BME of the knee who had either completed 30 sessions of HBOT or who had not received HBOT but had continued their follow-up in the hospital. Fifty-one patients were evaluated. Eight patients were excluded from the study: 5 of them had osteoarthritis, 2 had metastasis, and 1 had inflammatory arthritis.

All 43 BME patients were treated with 100 mg acetylsalicylic acid (ASA) daily and bisphosphonates (70 mg alendronate) once a week. They were asked to avoid weight-bearing activity. We divided the patients into 2 groups: those who had received HBOT (23 patients) and those who had not (20 patients).

Patients who had received HBOT did so by breathing 100% oxygen through masks under a 2.5-atmosphere compression in a multiplace chamber once a day, 5 days a week for 1 month.

**Figure 1.**
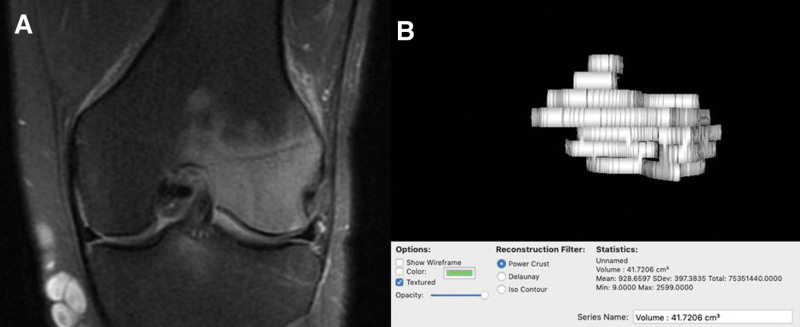
(A) MRI image of the knee with BME. (B) Measurement of BME 3D volume. BME = bone marrow edema, MRI = magnetic resonance imaging.

Patients’ original diagnostic MRIs were used as the control. MRIs taken after 3 months of HBOT were reviewed to measure the 3D volume of BMEs using HOROS version 3.3.6, a free and open-source medical image viewer (Fig. 1). Volumes of BMEs from the first and second scans were compared to determine whether the marrow had reduced in size after HBOT. This method enabled a quantitative evaluation of the effectiveness of HBOT.

### 2.1. Statistical analysis

Statistical analysis was undertaken via the Number Cruncher Statistical System 2007 program (Kaysville, UT). Descriptive statistical methods (mean, standard deviation, median, ratio, minimum, and maximum) were used when evaluating study data. The Wilcoxon test was used for comparisons of 2 periods. Chi-square analysis was used to determine the relationship between qualitative data. Mann–Whitney *U* analysis was used to determine the relationship between quantitative data. Significance was evaluated at *P* < .01 and *P* < .05 levels.

## 3. Results

A total of 43 patients, 21 female, and 22 male, were evaluated in our study. Their mean age was 48.1 years old. In the HBOT group, there were 23 patients: 13 female and 10 male. In the non-HBOT group, there were 20 patients: 8 female and 12 male. There was no statistically significant difference between the 2 groups regarding gender (*P* = .219) (Table 1).

**Table 1:** Gender and diagnosis comparisons between the 2 groups.

		Group		
		Hyperbaric oxygen treatment	Non-hyperbaric oxygen treatment	*P*
Gender	Women	13 (61.9%)	8 (38.1%)	.219*
	Men	10 (45.5%)	12 (54.5%)	
Diagnosis	Femoral condyle	14 (50%)	14 (50%)	.381*
	Tibial plato	9 (60%)	6 (40%)	

Chi-square test

* *P* < .01.

In the HBOT group, 14 patients had BME at the femoral condyles, and 9 had BME at the tibial plateau. In the non-HBOT group, 14 patients had BME at the femoral condyles and 6 at the tibial plateau. There was no statistically significant difference between the 2 groups regarding diagnoses (*P* = .381) (Table 1).

The mean age of the HBOT group was 50.7 years old (the range was 24–65). The mean age of the non-HBOT group was 44.9 years old (the range was 19–65). There was no statistically significant difference between the 2 groups regarding age (*P* = .168) (Table 2).

**Table 2:** Age comparison between the 2 groups.

		n	Mean ± SD	Min–max (median)	*P*
Age	Hyperbaric oxygen treatment	23	50.78 ± 10.66	24–65 (52)	.168*
	Non-hyperbaric oxygen treatment	20	44.9 ± 13.48	19–64 (47.5)	

SD = standard deviation.

Mann–Whitney *U* Test.

* *P* < .05.

In the HBOT group, the mean size of the BME lesion was 29.2 cm^3^, as measured on the diagnostic MRI. After HBOT, the mean size of the BME lesion was 19.19 cm^3^, as measured on a controlled MRI. The mean size of the BME lesions was reduced by nearly 35%. The reduction in lesion size after HBOT treatment was found to be statistically significant (*P* = .001) (Table 3).

**Table 3:** Comparision of lesion sizes in diagnoses and controlled MRIs of both groups.

		Diagnostic MRI	Controlled MRI	*P*
Hyperbaric oxygen treatment	Mean ± SD	29.2 ± 25.2	19.19 ± 25.35	.001*
	Min–max (median)	0.29–102,79 (23,36)	0–91.98 (8.7)	
Non-hyperbaric oxygen treatment	Mean ± SD	22.97 ± 20.36	21.94 ± 22.74	.247*
	Min–max (median)	0.3–79.93 (16.43)	0.5–79.12 (12.05)	

MRI = magnetic resonance imaging, SD = standard deviation.

Wilcoxon Test

* *P* < .01.

In the non-HBOT group, the mean size of the BME lesion was 22.97 cm^3^, as measured on the diagnostic MRI. After treatment without HBOT, the mean size of the BME lesion was 21.94 cm^3^, as measured on a controlled MRI. The mean size of BME lesions was reduced by nearly 5%. The reduction in lesion size in the non-HBOT group was not statistically significant (*P* = .247) (Table 3).

In the HBOT group, 2 patients developed middle ear barotrauma caused by changing air pressure in the cabin. Nevertheless, this side effect did not affect patients’ treatment. We did not see any other side effects related to ASA or bisphosphonates in either group.

## 4. Discussion and conclusions

The histopathologic pathway of BME is unknown, but several theories regarding its cause exist. One of these is that compromised blood support to the bone brings about an imbalance in its metabolism. Osteoblasts and osteoclasts maintain the balance of bone remodeling through osteoprotegerin (OPG), the receptor activator of the NF-kß (RANK) system, and the RANK ligand.^[7]^ Ischemia affects this system and leads to resorption.^[7]^

Avoiding weight-bearing activity can be suggested to prevent bone collapse, but this treatment choice is not curative.^[8]^ Nevertheless, patients were recommended to avoid weight-bearing activity and use double crutches.

Bisphosphonates are a suitable treatment option for osteoporosis. Alendronate, a type of nitrate-containing bisphosphonate, attaches to the bone surface. It prevents osteoclasts from destroying bone by inhibiting the mevalonate pathway and increasing osteoclastic apoptosis.^[9]^ Bisphosphonates are an appropriate treatment for BME.^[10,11]^ Alendronate (70 mg) was administered weekly to all patients without side effects.

ASA is a widely used non-steroidal anti-inflammatory drug. It affects cyclooxygenase-1 activity and inhibits thromboxane A2. It has an antithrombotic effect in addition to its anti-inflammatory effect.^[12]^ Compromised blood support is one of the hypotheses regarding the causes of BME, and ASA may prevent thrombosis in the capillary artery. It has been shown that ASA can be used in treating early-stage BME.^[12]^ Patients were given 100 mg ASA daily with no noticeable side effects.

HBOT affects OPG, RANK, and the RANK ligand system with increasing OPG levels. Increased OPG levels change bone metabolism to anabolism. Patients with early-stage avascular necrosis of the femoral head (ANFH) saw a reduction in pain and joint improvement in their MRI following HBOT.^[13]^ In addition, interleukin-1, tumor necrosis factor-a, and interleukin-6 affect bone metabolism with inflammation. HBOT reduces inflammation by lowering the levels of tumor necrosis factor-a and interleukin-6 in plasma. Under these conditions, patients with BME can see a reduction in pain.^[14]^ Another study compared pharmacological treatment and HBOT in ANFH, showing that treatment with HBOT accelerated recovery of function compared to pharmacological treatment.^[15]^ It has been shown that HBOT treats the early stages of ANFH successfully.

Studies show that HBOT therapy can not only be used in ANFH but also in knee BME.^[16,17]^ The results of HBOT in 37 patients with osteonecrosis of the knee were evaluated in a study, which determined that patient pain decreased and mobility increased with HBOT.^[16]^ In our study, HBOT patients with BME in their knees were compared against non-HBOT patients. The HBOT patients saw reductions in their BMEs over a shorter period.

The HBOT process could cause some side effects from the changing air pressure. It could cause middle ear barotrauma, paranasal sinus barotrauma, dental barotrauma, pulmonary barotrauma, ocular side effects, and pulmonary edema.^[18]^ One of the most frequently seen side effects of HBOT is middle ear barotrauma.^[18]^ This study showed only 2 mild middle ear barotraumas in patients that did not interfere with their treatment.

There are some limitations to our study. First, it was designed retrospectively and had a small sample size. It might have been more accurate if it had been designed prospectively and the sample size had been larger. Furthermore, the researcher who made the MRI measurements was aware of which treatments the patients had received, which could have created bias.

In conclusion, HBOT is an effective treatment option for BME. We quantitatively measured faster healing when HBOT was used for BME of the knee. There were no significant side effects.

## Author contributions

**Data curation:** Burak Ozturan.

**Investigation:** Burak Ozturan.

**Methodology:** Burak Ozturan, Muhlik Akyuerek.

**Writing – original draft:** Burak Ozturan.

**Writing – review & editing:** Burak Ozturan, Muhlik Akyuerek.
